# Humic Acid Fertilizer Improved Soil Properties and Soil Microbial Diversity of Continuous Cropping Peanut: A Three-Year Experiment

**DOI:** 10.1038/s41598-019-48620-4

**Published:** 2019-08-19

**Authors:** Yan Li, Feng Fang, Jianlin Wei, Xiaobin Wu, Rongzong Cui, Guosheng Li, Fuli Zheng, Deshui Tan

**Affiliations:** 1 Institute of Agricultural Resources and Environment, Shandong Academy of Agricultural Sciences, Ji’nan, 250100 China; 2 Key Laboratory of Wastes Matrix Utilization, Ministry of Agriculture, Ji’nan, 250100 China; 3 Shandong Provincial Key Laboratory of Plant Nutrition and Fertilizer, Ji’nan, 250100 China; 4 Institute of Plant Protection, Shandong Academy of Agricultural Sciences, Ji’nan, 250100 China; 5 Shandong Rice Research Institute/Hydrobiology Research Center, Shandong Academy of Agricultural Sciences, Ji’nan, 250100 China

**Keywords:** Environmental impact, Microbial ecology

## Abstract

Although humic acid has been demonstrated to improve the quality of some soil types, the long-term effects of humic acid on soil under continuous cropping peanut are not fully understood. This study aimed to investigate the continuous effects of humic acid on the physicochemical properties, microbial diversity, and enzyme activities of soil under continuous cropping peanut. In this study, a three-year consecutive experiment of cropping peanut was conducted in the North China Plain. In addition to the equal nitrogen, phosphorus, and potassium inputs, humic acid treatment was applied with inorganic fertilizers. Compared with control experiments, humic acid increased the yield and quality of continuous cropping peanut. To elucidate the mechanism of humic acid affecting the soil quality, various soil quality indicators were evaluated and compared in this study. It was found that humic acid increased soil nutrient contents, including the total soil nitrogen, total phosphorus, total potassium, available nitrogen, available phosphorus, available potassium, and organic matter contents, which exhibited the maximum effect in the third year. Meanwhile, the urease, sucrase, and phosphatase activities in the soil significantly increased after treated with humic acid, of which the maturity period increased most significantly. The same results were observed for three consecutive years. Microbial diversity varied considerably according to the high throughput sequencing analysis. Specifically, the number of bacteria decreased while that of fungi increased after humic acid treatment. The abundance of Firmicutes in bacteria, Basidiomycota, and Mortierellomycota in fungi all increased, which have been reported as being beneficial to plant growth. In contrast, the abundance of Ascomycota in fungi was reduced, and most of the related genera identified are pathogenic to plants. In conclusion, humic acid improved the yield and quality of continuous cropping peanut because of improved physicochemical properties, enzymatic activities, and microbial diversity of soil, which is beneficial for alleviating the obstacles of continuous cropping peanut.

## Introduction

Peanut (Arachis hypogaea L.) is an important economic and oil crop in China. In recent years, the production scale of peanut has continued to increase, and the peanut production areas in China are relatively concentrated. Due to the relatively high planting benefits of peanut and the limitation of planting soil, continuous cropping of peanut is a serious phenomenon^1^, which is a primary cause for the yield and quality loss of peanut^2^. In addition to the deterioration of soil physical and chemical properties, the imbalance of soil microbial flora is also an important cause^3–5^. Therefore, we have adopted a series of measures to alleviate the continuous cropping obstacles of peanuts based on the mechanism of continuous cropping obstacle formation.

Studies have shown that the application of organic fertilizer can improve soil fertility, reduce soil-borne diseases, improve microbial flora structure, thus alleviating the continuous crop obstacle^6^. Humic acid is a kind of organic matter produced and accumulated by animal and plant remains through the decomposition and transformation of microorganisms with a series of geochemistry processes. Combined with various inorganic fertilizers, humic acid can improve the soil quality, enhance fertilizer utilization rate^7,8^, and promote crop yield and quality^9–11^. Most of the current research focuses on the physicochemical state of humic acid to improve the soil quality while few studies have been conducted to investigate the influence of humic acid on soil microbes. Therefore, we focused on the impact of humic acid on soil microbials. Soil microbes are the most active part of the soil, which is the driving force for material transformation and nutrient cycling in the soil and is involved in various processes, such as soil organic matter decomposition, humus formation, soil nutrient conversion, and circulation^12,13^. Many studies have revealed that the soil microbial community structure was affected by soil treatment conditions. Moreover, the application of chemical fertilizers^14^, pesticide application^15^, and different tillage systems^16^ have been demonstrated to impact soil microbial community structure. Due to the rapid response of microorganisms to environmental changes, in addition to soil nutrient content and physicochemical properties, microbial community structure changes are considered as effective biomarkers for soil conditions and land quality changes^17,18^. To study the influnence of humic acid on soil microbials can reflect the effect of humic acid on soil improvement.

In recent years, high-throughput sequencing technology has been developed for determining microbial diversity. The 16S/ITS rRNA gene fragments obtained directly from soil samples were sequenced by employing this method to study the microbial community structure and diversity in soil samples. Since this method has various advantages, including high flux, a large amount of information, simple operation, low cost, and short time, it has been widely used in the study of soil microbial diversity with remarkable outcomes^19,20^.

Given the unclear effects of humic acid on the physical and chemical properties and microbial diversity of continuous peanut rhizosphere soil, we investigated the effects of humic acid on soil nutrient content, enzyme activity, and other physical and chemical properties in this experiment. On the other hand, high-throughput quantitative sequencing technology was employed to study the effects of humic acid on the diversity of bacteria and fungi. This study clarified the effect of humic acid on the physical and chemical properties and soil microbial community structure of continuous cropping soil, and elucidated the mechanism that humic acid can alleviate peanut continuous cropping obstacle and improved the yield and quality of peanut. It provides theoretical basis for adopting new measures to alleviate the peanut continuous cropping obstacle in agricultural production.

## Results

### Peanut yield and grain quality

The results of the three-year study (Table 1) showed that peanut pod number, pod weight, and peanut per pot yield with the humic acid treatment (HA) significantly increased annually and reached the highest in 2018. The yield of peanut per pot treated with HA increased by 78.29% compared with the control (C) in 2018. The content of fat and protein also increased after treated with HA. However, HA treatment decreased the content of oleic acid but increased the content of linoleic acid, which leads to a decreased O/L.

**Table 1:** Peanut yield and grain quality.

Years	Treatment	Pod NO.	Pod weight (g)	Yield (g/pot)	Fat (%)	Protein (%)	Oleic acid (%)	Linoleic acid (%)	O/L
2016	C	12.17d	18.90d	115.85d	41.52b	30.00b	47.40a	30.43b	1.56a
	HA	16.33c	28.09c	166.76c	43.65a	31.30a	46.21c	31.25ab	1.48b
2017	C	16.33c	20.13d	120.40d	41.67b	31.70a	46.86b	30.48b	1.54a
	HA	18.83b	30.37b	181.97b	43.53a	31.39a	46.19c	31.82a	1.45b
2018	C	16.00c	18.85d	115.22d	41.73b	30.86ab	47.55a	30.28b	1.57a
	HA	20.50a	35.65a	205.42a	43.95a	31.81a	46.24c	31.19ab	1.48b

### Chemical properties of peanut rhizosphere soil

The chemical properties of the rhizosphere soil under different treatment conditions are shown in Table 2. The soil pH of HA significantly decreased during the three years (2016–2018). After treated with humic acid, the soil total nitrogen (TN), total phosphorus (TP), total potassium (TK), available N (AN), available P (AP), available K (AK) and the content organic matter (OM) content significantly increased from the second years and reached the highest level by the third year. The contents of TP, AP, and AK, did not significantly change in 2016, and after that, increased continuously, and reached the highest in 2018. The content of TN decreased in 2016 compared with the control and then increased from 2017. The content of TK decreased slightly in 2016. However, it was higher than that in 2018.

**Table 2:** Changes of the chemical properties of tested soil sample for three years tests.

Years	Treatment	Total N (g/kg)	Total P (g/kg)	Total K (g/kg)	Available N (mg/kg)	Available P (mg/kg)	Available K (mg/kg)	Organic matter (g/kg)	pH
2016	C	1.15d	0.41b	15.80b	51.00d	31.94b	91.75c	10.22c	8.13ab
	HA	1.11e	0.41b	15.10c	51.22 cd	32.19b	92.83c	10.67c	8.09b
2017	C	1.18bc	0.42b	16.07b	51.45 cd	31.87b	91.93c	10.54c	8.15ab
	HA	1.19b	0.44ab	16.23b	52.67b	36.43a	95.52b	12.87b	8.09b
2018	C	1.16 cd	0.41b	16.07b	51.83c	32.24b	91.70c	10.46c	8.18a
	HA	1.22a	0.46a	17.40a	55.60a	36.77a	98.83a	14.29a	8.13ab

### Enzyme activity

**Figure 1:**
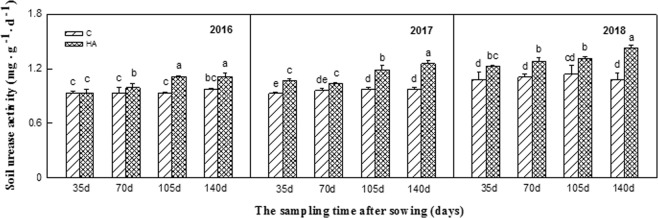
Soil urease activities during peanut growth period in 2016–2018. Different lowercase letters above the bar indicate significant difference among the treatments (P < 0.05). HA: humic acid treatment, C: control treatment.

**Figure 2:**
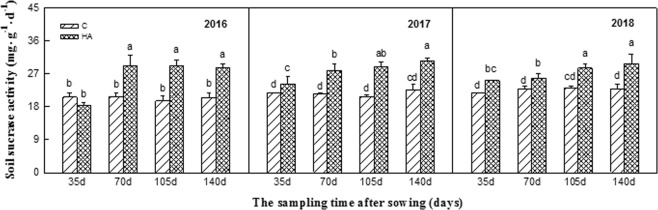
Soil sucrase activities during peanut growth period in 2016–2018. Different lowercase letters above the bar indicate significant difference among the treatments (P < 0.05). HA: humic acid treatment, C: control treatment.

**Figure 3:**
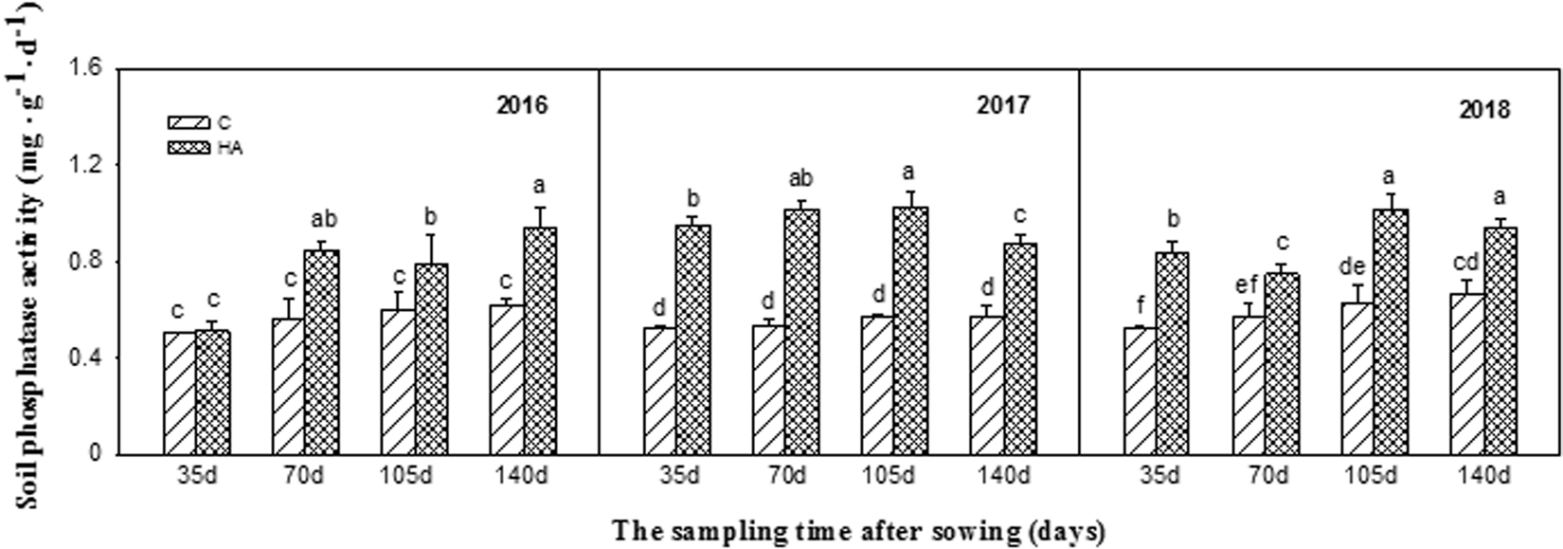
Soil phosphatase activities during peanut growth period in 2016–2018. Different lowercase letters above the bar indicate significant difference among the treatments (P < 0.05). HA: humic acid treatment, C: control treatment.

Soil urease, sucrase, and phosphatase activities all significantly increased after HA treatment from 2016–2018. Particularly in 2018, the effect was the most significant. The soil urease activity continuously increased during the peanut growth period and reached the highest in the maturing stage. Compared with the control, the soil urease activity increased by 13.22%, 15.32%, 15.32%, and 31.98% after sowing for 35 d, 70 d, 105 d, and 140 d, respectively (Fig. 1). Similarly, the soil sucrase activity continuously increased during the peanut growth period and reached the highest in the 105 d and 140 d after sowing. Compared with the control, the soil sucrase activity increased by 15.92%, 12.21%, 23.07%, and 29.90% after sowing for 35 d, 70 d, 105 d, and 140 d, respectively (Fig. 2). The growth trend of soil phosphatase activity was consistent with that of the sucrose, which reached the highest in the 105 d and 140 d. Compared with the control, the soil phosphatase activity increased by 60.10%, 30.15%, 60.43%, and 40.44% after sowing for 35 d, 70 d, 105 d, and 140 d, respectively (Fig. 3).

### Microbial community diversity

The microbial diversity from HA and C was characterized by partial 16S and ITS rRNA gene sequencing obtained from DNA directly extracted from soil samples of continuous cropping peanut treated with humic acid. In total, 66620 and 80210 high-quality reads were analyzed for bacteria and fungi, respectively. The coverage of bacterial and fungi clone libraries was all above 97%, indicating that the major of the biofilm diversity in the clone libraries was detected. In addition, the rarefaction curves generated from our clones reached the asymptote (Figs S1 and S2), suggesting that the diversity in the libraries was representative of the community, and there was no need for a further sampling of more clones.

### Microbial taxonomic composition

Relative abundances of bacterial and fungal taxa were examined at the phyla and class levels to determine whether there were any significant shifts in the composition of the microbial communities according to humic acid treated samples.

**Figure 4:**
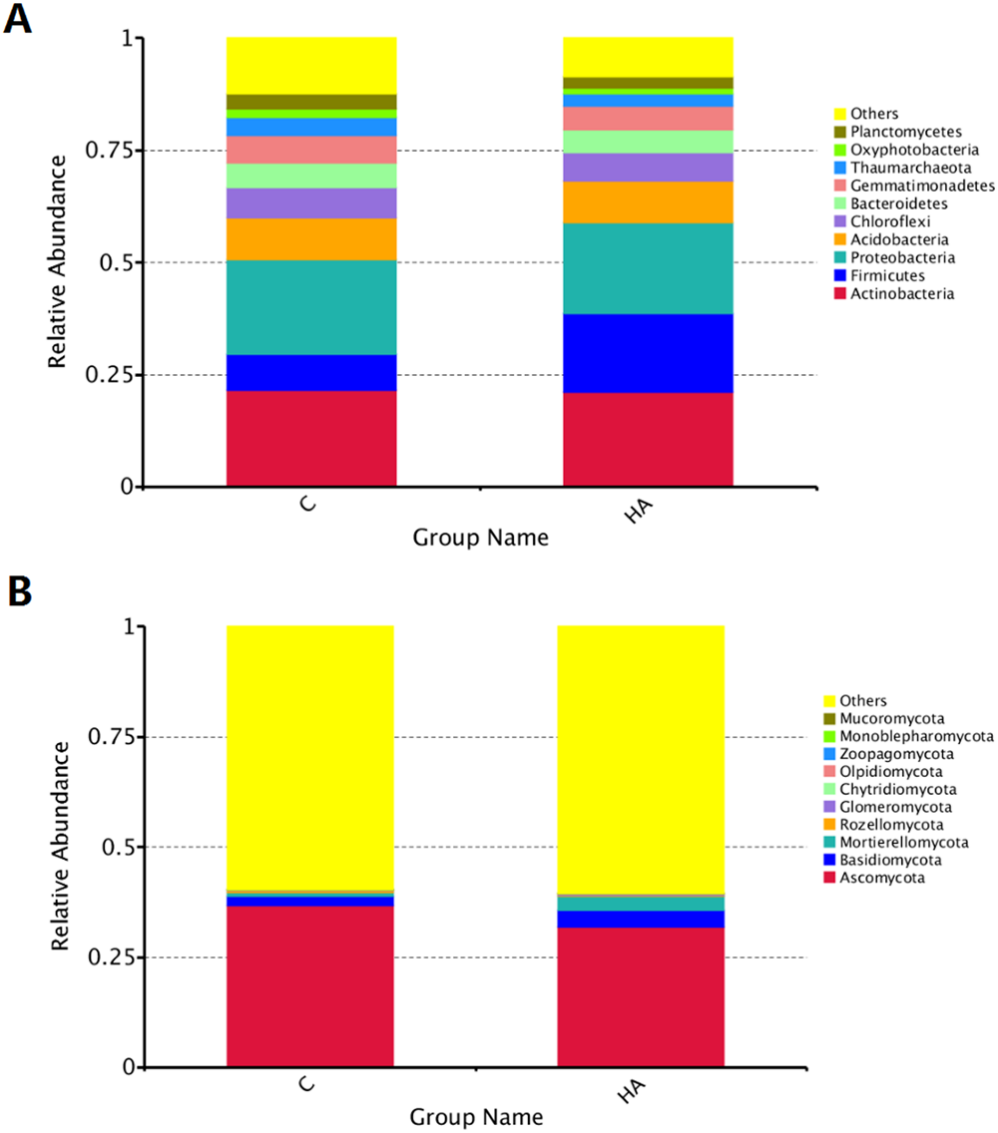
Abundance of bacteria (**A**) and fungi (**B**) phyla in soil sample in 2018. Only OTUs with an indicidence >1% in at least one sample are shown. HA: humic acid treatment, C: control treatment.

As shown in Fig. 4, the high phyla in bacteria were Actinobacteria, Firmicutes, Proteobacteria, Acidobacteria, Chloroflexi, Bacteroidetes, which account for approximately more than 75% of the total biodiversity in each sample. After humic acid treatment, the abundance of Firmicutes increased by 1.16 fold while other phylum decreased by a range of 0.43% to 32.60% compared with the control. Among them, Oxyphotobacteria and Thaumarchaeota were reduced by 30.56% and 32.60% (Fig. 4A), respectively. In fungi, their abundances were all low. Specifically, the Ascomycota, Basidiomycota, and Mortierellomycota accounted for approximately 39% of the total biodiversity in each sample, while others accounted for approximately 60%. After humic acid treatment, the Ascomycota decreased by 13.07%. In contrast, Basidiomycota and Mortierellomycota increased by 81.95% and 3.16 fold, respectively, compared with the control (Fig. 4B).

The microbial diversity was also analyzed at a deep taxonomic level. The identification of operational taxonomic units (OTUs) at the class level is illustrated in the thermography shown in Figs S3 and S4. As expected, the hierarchical clustering analysis based on taxa and samples grouped humic acid treated and control soil.

### Microbial biomarkers and core evaluation

**Figure 5:**
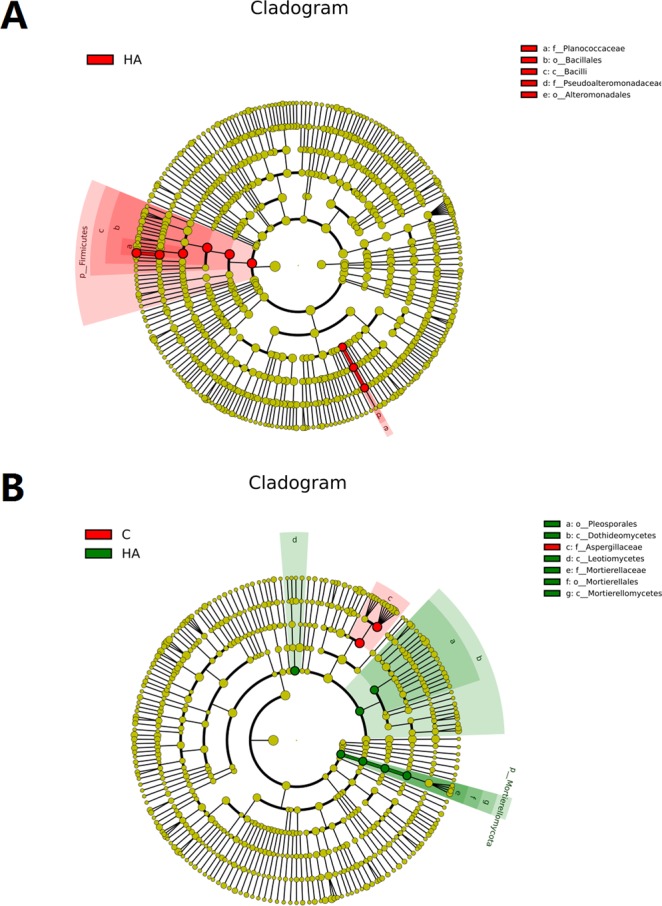
LEfSe cladograms showing taxa with different abundance values (LDA score > 2; p < 0.05) in bacteria (**A**) and fungi (**B**). HA: humic acid treatment, C: control treatment. Each ring represents the next taxonomic level (phylum, class, family, genus and species).

From phyla to genera, the two samples showed their specific influence on bacterial and fungal compositions (LDA > 2; P < 0.05). In bacterial, the abundances of Planococcaceae, Bacillales, and Pseudoalteromonadaceae were significantly different after HA treatment (Fig. 5A). In fungi, the abundances of Pleosporales, Dothideomycetes, Leotiomycetes, Motierellaceae, Mortierellales, Mortierellomycetes, and Aspergillaceae also changed significantly (Fig. 5B).

**Figure 6:**
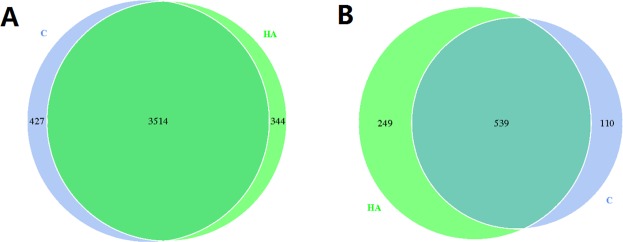
Venn diagrams of shared bacteria (**A**) and fungi (**B**) biomarkers among soil samples. HA: humic acid treatment, C: control treatment. Each circle represents a set of samples. Different color represents different sample. The numbers in the overlap region represent the number of OTU shared by all samples. The numbers on the non-overlap region represent the number of OTU unique to the sample.

To identify potential bacterial and fungal biomarkers regardless of the different treatment soil samples, Venn diagram analysis was carried out. As showed in Fig. 6A, the sum of total observed OTUs in bacteria was 4285, including 3514 OTUs that is common to all treatments. Specific OTUs were 427 in the control sample, and 344 in HA treated sample. It was evident that humic acid treatment not only reduced the total OTUs numbers but also reduced the specific OTUs. In fungi, the sum of total observed OTUs was 898, including 539 common OTUs to all treatments, 110 specific OTUs in control, and 249 specific OTUs in HA treatment. As opposed to bacteria, humic acid treatment not only increased the total OTUs numbers but also increased the specific OTUs (Fig. 6B).

## Discussion

This study has revealed that humic acid combined with inorganic fertilizer could improve the yield and quality of continuous cropping peanuts. The same effect was also observed on other crops^21–25^. However, the previous study was mostly a one-year test result, and the experiment in this study was based on the application of humic acid in three consecutive years. This study also indicated that the effect of humic acid on improving yield and quality of continuous cropping peanut was enhanced with increasing HA application time.

To study the mechanism that humic acid can alleviate peanut continuous cropping obstacles and improve the yield and quality of continuous cropping peanut, we conducted this study mainly in the following three aspects. (1) the effect of humic acid on soil nutrient content; (2) the effect of humic acid on soil enzyme activity; and (3) the effect of humic acid on soil microbial diversity.

Firstly, the HA treatment increased not only the soil TN, TP, TK contents and AN, AP, AK contents but also the OM content. Furthermore, the nutrition content increased with the extension of years (Table 2). This result indicated that the addition of humic acid could enhance the nutrient content of the soil under continuous cropping peanut, especially increase the content of soil organic matter, which is beneficial to the improvement of farmland soil^26^. Extensive studies have indicated that the increase in the soil OM content improved soil ventilation and aggregation, thus benefiting crop growth and yield^27^. The application of humic acid in the soil growing tobacco could generate contaminated tobacco soil and improve the soil ecological environment^28^. This study confirmed the long-term effect of humic acid on improving soil nutrient status through three consecutive years of test results.

Secondly, the changes in soil urease, sucrase, and alkaline phosphatase activities were measured after the treatment of humic acid in this experiment. It was found that humic acid could increase the activities of these three enzymes during the whole growth period of continuous cropping peanuts, and the effect was more significant with the extension of application time (Figs 1–3). Soil enzymes are the products of animal and plant residue decomposition, plant root exudation, and soil microbial metabolism in the soil, which are a type of special substances with biochemical catalytic activity and involved in many important biochemical processes in soil^29^. The level of soil enzyme activity can represent the exuberant degree of material metabolism in the soil, which also can reflect the nutrient absorption and utilization and growth of crops to a certain extent. The activity of soil enzyme is an important indicator of soil fertility, soil quality, and soil health^30,31^. This study indicated that humic acid could increase the urease activity of the soil, which is consistent with Wang Libin’s research result that humic acid could significantly increase urease activity in sorghum soil^32^. However, some studies have revealed that humic acid formed a complex with urea, thus inhibiting urease activity^33^. The reason for the different results may be attributed to the different soil basic conditions. In this study, humic acid increased the activities of sucrase and phosphatase in the soil, and the same results were obtained by applying humic acid to other crops^34^. This is because humic acid is rich in organic matters that can improve the physical and chemical properties of soil, thereby increasing soil enzyme activity. Therefore, the combination of humic acid and inorganic fertilizer is beneficial to enhance the quality of continuous cropping peanut soil, improve soil fertility, and alleviate peanut continuous cropping obstacles.

In this study, high-throughput sequencing was employed to study the changes of microbial diversity in the soil under continuous cropping peanut after addition of humic acid to inorganic fertilizers. Rhizosphere soil microbes are an important part of rhizosphere micro-ecology, which play an important role in organic matter decomposition, nitrogen fixation, soil nutrient transformation, and plant growth and development in rhizosphere soil, but they are very sensitive to environmental changes^35^. Fertilization can significantly change the soil microbial community structure^36,37^, long-term and balanced fertilization enhances soil microbial biomass, carbon and nitrogen contents, and functional activities^38^. This study revealed that after the application of humic acid, the number and structure of bacteria and fungi in continuous cropping peanut soil changed, in which the number and types of bacteria decreased while those of fungi increased (Figs 4–6). Previous studies have shown that after the addition of organic matters to the soil, the number of beneficial bacteria increased while the number of harmful bacteria decreased^39,40^, which is consistent with the results of this study. After HA treatment, the abundances of Firmicutes in bacteria, Basidiomycota, and Mortierellomycota in fungi all increased, which have been reported as being beneficial to plant growth^41–43^. However, the abundance of Ascomycota in fungi decreased, and most of the related genera identified are pathogenic to plants^5^. In addition, the application of alkaline fertilizer to acidic soil changed the acid-base environment of the original microorganisms in the soil, which led to the number of the most abundant actinomycetes in the soil increased^44^. Since the humic acid used in this study is acidic, which can lower the pH of the soil in the field, it is speculated that the decrease in pH value is one of the causes that reduce the number of bacteria. In conclusion, the addition of humic acid can change the microbial community structure of continuous cropping peanut soil, making the soil more conducive to peanut growth and thereby alleviating peanut continuous cropping obstacles.

In conclusion, it can be concluded from this study that the addition of humic acid to inorganic fertilizers can increase the yield and quality of continuous cropping peanuts and alleviate the continuous cropping obstacles of peanut, which is mainly due to the following several reasons. Firstly, humic acid changes the soil nutrient content, which not only increases the total nitrogen, total phosphorus, total potassium content of the soil, but also increases the contents of alkali nitrogen, available phosphorus, and available potassium, thus enabling peanut to absorb more nutrients. At the same time, it also increases the soil organic matter content, which is conducive to the long-term sustainable use of soil. Secondly, humic acid changes the activities of sucrase, urease, and phosphatase in the soil, and enhances the metabolism of substances in the soil. The change in enzyme activity also affects soil microbial activity. Thirdly, humic acid changes the community structure of soil microorganisms, increasing beneficial microorganisms, and reducing harmful microorganisms, which is favorable for peanut growth. Since soil is a complex environment, after adding humic acid, various factors affect each other, which synergistically improve the soil quality of continuous cropping peanuts and enhance nutrient utilization, thus achieving the effect of enhanced peanut yield and quality. This study focuses on the effect of humic acid on the soil under continuous cropping peanut, but its impact on continuous cropping peanut needs further research.

## Material and Methods

### Experimental sites and soil

The field experiment was conducted in the Drinking Horse Spring experimental farm at the Shandong Academy of Agricultural Sciences, located in Jinan, Shandong Province (117°5′E, 36°43′N). The mean annual temperature is 14.7 °C with an annual sunshine duration of 2616.8 h and mean annual precipitation of 671.1 mm. The experiment was conducted from the years 2016 to 2018. The experimental crop is peanut “Huayu 22”. Six strain peanuts were planted in each pot with 20 kg soil, which was collected from the topsoil (0–20 cm) under continuous cropping peanut for five years. Each treatment has 30 pots for the experiment. Major soil properties before the experiment are listed in Table 3.

**Table 3:** Physical and chemical properties of tested soil sample.

Soil	Total N (g/kg)	Total P (g/kg)	Total K (g/kg)	Available N (mg·kg^−1^)	Available P (mg·kg^−1^)	Available K (mg·kg^−1^)	Organic matter (g·kg^−1^)	pH
Continuous cropping for 5 years	1.05	0.38	15.46	47.97	23.15	87.43	10.11	8.32

### Experimental treatment and fertilization

Two treatment groups were set up in this experiment, namely inorganic fertilizers (control, C), and humic acid fertilizer (HA). Three replicates were performed for each treatment. The irrigation time, frequency, and quantity were identical among the two treatments. The treatment for the C and HA groups were based on local farming habits. Specifically, 58.7 kg/ha of urea, 33.3 kg/ha of triple superphosphate and 37.5 kg/ha of potassium chloride were applied as nitrogen, phosphate and potassium fertilizers, respectively, which equal to 27.0 kg/ha of pure nitrogen, 15.0 kg/ha P_2_O_5_, and 22.5 kg/ha K_2_O, respectively. These fertilizers were applied as a one-time base fertilizer into the topsoil (0–20 cm). The HA was added 1000 kg/ha humic acid (from the Dalian Jiucheng Products Company (Dalian, China), with humic acid 75%), which was applied with the base fertilizer.

### Soil sampling and analysis

Before planting peanut, 0–20 cm soil was randomly sampled by the five-point composite method throughout the experimental site to measure the basic physical and chemical properties of the soil before the start of the experiment. After harvesting, the major properties of rhizosphere soil was measured from 2016 to 2018. Soil samples were stored in Institute of Agricultural Resources and Environment, Shandong Academy of Agricultural Sciences, Ji’nan, China. The pH was measured by using a pH meter/potentiometer (PHBJ-260 portable pH meter, 0.01 pH resolution; Shanghai Precision & Scientific Instrument, Shanghai, China). The soil organic matter (OM) content was measured by the potassium dichromate external heating method. The contents of total nitrogen (TN) and total phosphorus (TP) were determined by continuous flow analysis (AutoAnalyzer 3, sensitivity 0.001 AUFS; Bran & Luebbe, GmbH, Norderstedt, Germany). The total potassium (TK) was quantified by using inductively coupled plasma-atomic emission spectrometry (ICPS-7500, Shimadzu, Japan). The soil available nitrogen (AN), available phosphorus (AP), and available potassium (AK) were determined by alkaline hydrolysis diffusion method, molybdenum antimony colorimetry, and flame photometry method, respectively. After sowing 35 d, 70 d, 105 d, and 140 d, enzyme activities in the soil were measured separately. The activities of urease, sucrase, and alkaline phosphatase in the soil were determined by using phenol-sodium hypochlorite colorimetric method, 3, 5-dinitro salicylic acid colorimetric method, and disodium phenyl phosphate colorimetric method, respectively.

### Peanut yield and quality analysis

In the study of a three-year period, the average per pot yield was measured by harvesting ten pots of peanut from each treatment that were air-dried and weighed. The protein and fatty acid contents in peanut were determined by near-infrared spectroscopy method with the VECTOR 22/N Fourier transform near-infrared spectrometer (BRUKER, Germany) and HP6820 gas chromatograph (Agilent, USA).

### DNA extraction and Illumina sequencing

Total genomic DNA was extracted using the Power Soil kit (MO BIO Laboratories, Carlsbad, CA, USA) following the manufacturer’s instructions. The specific primers of bacterial 16S rRNA gene and the partial ITS region of fungi were used for the amplification. The PCR reactions, quality control, and purification processes were followed the instructions of Yao *et al*.^45^. A library was constructed, and all sequences were generated with the Illumina’s MiSeq platform (2 × 250) using paired-end reads. All steps mentioned above were conducted at the Beijing Novogene Technology Co. LTD, Beijing, China.

### Bioinformatics and data analysis

According to the Barcode sequence and the PCR amplification primer sequence, each sample data was separated from the offline data. After the Barcode and the primer sequence were truncated, the reads of each sample were spliced by using FLASH^46^, and the obtained splicing sequence was the original Tags data (Raw Tags). Raw tags obtained by splicing need to undergo a strict filtering process^47^ to obtain high-quality tags data (Clean Tags) according to Qiime^48^ tags quality control process. The processed tags need to be processed to remove the chimeric sequence, and the tags sequence^49^ was compared with the database (Gold database, http://drive5.com/uchime/uchime_download.html) to detect the chimeric sequence and finally eliminated the chimera sequence^50^, thus yielding the final effective data (Effective Tags).

All the effective tags of all samples were clustered by using Uparse software (Uparse v7.0.1001, http://drive5.com/uparse/)^51^. By default, the sequences were clustered into OTUs with 97% consistency, and the representative sequence of OTUs was selected. According to its algorithm principle, the screened sequence with the highest frequency in OTUs was considered as the representative sequence of OTUs. The data was deposited to figshare. The data access link was https://figshare.com/s/e3f6d73098e15cd8468d.

The Qiime software (Version 1.7.0) was used to calculate the Chao, Shannon, Simpson, and ACE indicators, and the R software (Version 2.15.3) was used to draw the dilution curve. The Alpha diversity index analysis and the Beat diversity index were conducted. The petaline graphs, thermography, and principal component analysis graphs were obtained by using R software and processed by using Excel and SPSS. All steps mentioned above were completed at the Beijing Novogene Technology Co. LTD, Beijing, China. Large scale data was deposited by Institute of Agricultural Resources and Environment, Shandong Academy of Agricultural Sciences, Ji’nan, China.

### Statistical analyses

All data were analyzed using the SPSS software (IBM Corporation, Armonk, NY, USA), and the results were expressed as the arithmetic mean value ± standard deviation. The differences in the means were compared by the least significant difference (LSD) test at P < 0.05.

## Supplementary information

Supplementary figure 1, Supplementary figure 2, Supplementary figure 3, Supplementary figure 4
