# Supplementary material for: Humic Acid Fertilizer Improved Soil Properties and Soil Microbial Diversity of Continuous Cropping Peanut: A Three-Year Experiment
Source: Sci Rep. 2019 Aug 19;9:12014. doi: 10.1038/s41598-019-48620-4 (PMC6700118; doi:10.1038/s41598-019-48620-4)
Supplement: Supplementary file 1 — Supplementary figure 1, Supplementary figure 2, Supplementary figure 3, Supplementary figure 4 [file 41598_2019_48620_MOESM1_ESM.doc]

Humic Acid Fertilizer Improved Soil Properties and Soil Microbial Diversity of Continuous Cropping Peanut: A Three-Year Experiment

Yan Li^1, 2, 3^, Feng Fang^4^, Jianlin Wei^1^, Xiaobin Wu^5^, Rongzong Cui^1^, Guosheng Li^1^, Fuli Zheng^1^, Deshui Tan^1, 2*^

^1^Institute of Agricultural Resources and Environment, Shandong Academy of Agricultural Sciences, Ji’nan 250100, China; ^2^Key Laboratory of Wastes Matrix Utilization, Ministry of Agriculture, Ji’nan 250100, China; ^3^Shandong Provincial Key Laboratory of Plant Nutrition and Fertilizer, Ji’nan 250100, China; ^4^Institute of Plant Protection, Shandong Academy of Agricultural Sciences, Ji’nan 250100, China. ^5^Shandong Rice Research Institute/Hydrobiology Research Center, Shandong Academy of Agricultural Sciences, Ji’nan 250100, China. Correspondence and requests for materials should be addressed to Y. L. (email: liyan1008@163.com).

**Feng Fang was the Co-first author; Deshui Tan was the Corresponding author.**


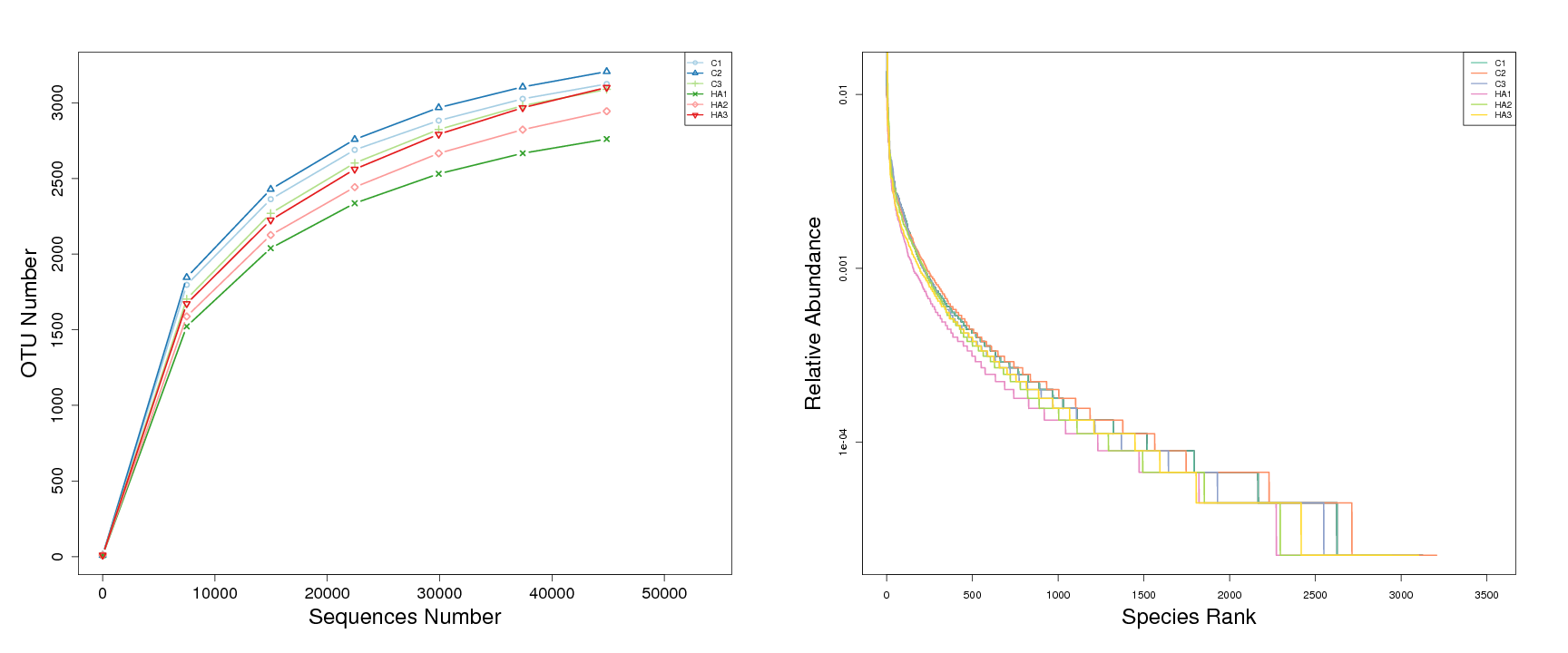


**Supplementary figure 1.** Rarefaction curves of bacteria in humic acid treated (HA) and control (C) soil samples.


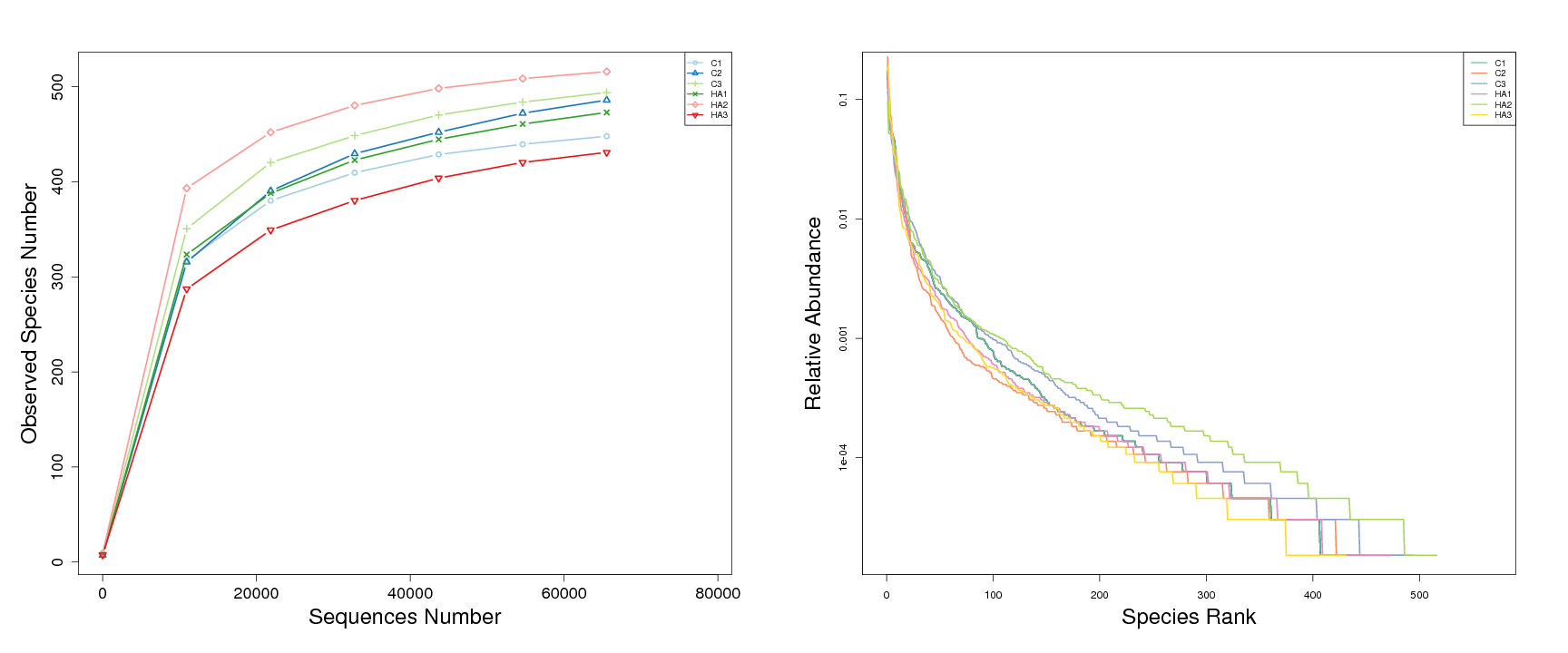


**Supplementary figure 2.** Rarefaction curves of fungi in humic acid treated (HA) and control (C) soil samples.





**Supplementary figure 3.** Heatmap representing bacteria taxa identified in humic acid treated (HA) and control (C) soil samples.





**Supplementary figure 4.** Heatmap representing fungi taxa identified in humic acid treated (HA) and control (C) soil samples.
